# Method for Augmenting Side-Scan Sonar Seafloor Sediment Image Dataset Based on BCEL1-CBAM-INGAN

**DOI:** 10.3390/jimaging10090233

**Published:** 2024-09-20

**Authors:** Haixing Xia, Yang Cui, Shaohua Jin, Gang Bian, Wei Zhang, Chengyang Peng

**Affiliations:** Department of Oceanography and Hydrography, Dalian Naval Academy, Dalian 116018, China; a1466448779@163.com (H.X.); jsh_1978@163.com (S.J.); trighosts@163.com (G.B.); 18590361852@163.com (W.Z.); 18908414801@163.com (C.P.)

**Keywords:** sample amplification, side-scan sonar, background image, convolutional attention mechanism, BCEL1loss function

## Abstract

In this paper, a method for augmenting samples of side-scan sonar seafloor sediment images based on CBAM-BCEL1-INGAN is proposed, aiming to address the difficulties in acquiring and labeling datasets, as well as the insufficient diversity and quantity of data samples. Firstly, a Convolutional Block Attention Module (CBAM) is integrated into the residual blocks of the INGAN generator to enhance the learning of specific attributes and improve the quality of the generated images. Secondly, a BCEL1 loss function (combining binary cross-entropy and L1 loss functions) is introduced into the discriminator, enabling it to focus on both global image consistency and finer distinctions for better generation results. Finally, augmented samples are input into an AlexNet classifier to verify their authenticity. Experimental results demonstrate the excellent performance of the method in generating images of coarse sand, gravel, and bedrock, as evidenced by significant improvements in the Frechet Inception Distance (FID) and Inception Score (IS). The introduction of the CBAM and BCEL1 loss function notably enhances the quality and details of the generated images. Moreover, classification experiments using the AlexNet classifier show an increase in the recognition rate from 90.5% using only INGAN-generated images of bedrock to 97.3% using images augmented using our method, marking a 6.8% improvement. Additionally, the classification accuracy of bedrock-type matrices is improved by 5.2% when images enhanced using the method presented in this paper are added to the training set, which is 2.7% higher than that of the simple method amplification. This validates the effectiveness of our method in the task of generating seafloor sediment images, partially alleviating the scarcity of side-scan sonar seafloor sediment image data.

## 1. Introduction

With the continuous development and utilization of marine resources, research on seafloor sediment environments has gained increasing attention. Side-scan sonar technology, as a crucial underwater detection method, plays a significant role in marine surveys, underwater archeology, and seafloor geological studies [1,2,3,4]. Side-scan sonar systems transmit sound waves and record their reflections to generate seafloor sediment images, which are essential for understanding seabed topography, detecting underwater targets, and assessing changes in marine environments [5,6].

However, traditional side-scan sonar seafloor sediment images are often constrained by factors such as underwater environmental complexity, imaging resolution limitations, and insufficient lighting conditions. These issues result in noise, blurriness, and occlusions in the images, limiting their usability and application scope. Moreover, acquiring datasets of seafloor sediment images is challenging due to high labeling costs and a lack of diversity and quantity of data samples [7,8,9]. Therefore, effectively enhancing and augmenting side-scan sonar seafloor sediment images to improve their quality and informativeness have become current research hotspots. With the advancement and widespread adoption of deep learning technologies, as well as the increasing demand in marine research, deep learning-based object detection methods far surpass traditional machine learning approaches, garnering extensive attention in underwater detection fields [10,11,12,13]. The automatic identification and classification of seafloor sediment images also hold significant importance in marine geological surveys and seabed resource exploration. There are studies on methods for augmenting images of underwater targets obtained using side-scan sonar, such as the method designed by Tang Yulin et al. [14] based on CSLS-CycleGAN for augmenting high-quality samples of zero- and small-sample underwater representative targets, methods for augmenting samples of side-scan sonar sediment images are lacking.

Currently, the mainstream method for image augmentation involves using generative adversarial networks (GANs) for image generation, with most networks requiring large datasets for training. However, there is a significant scarcity of side-scan sonar images corresponding to certain types of sediment in existing datasets. For instance, Quanyin Zhang et al. [15] encountered the problem of having only one sample point for coarse sand in their sediment classification study, merging coarse sand with fine sand into the sand category. Therefore, there is a need to design GANs capable of augmenting small samples to address the scarcity of side-scan sonar seafloor sediment image data. In 2019, Assaf Shocher et al. [16] proposed the INGAN network, which is suitable for training on a single input image and learning block distributions within it to synthesize numerous new natural images of different sizes. However, using the INGAN network to train side-scan sonar seafloor sediment images tends to generate unrealistic images. Thus, to enhance the quality of the generated images, this paper proposes an improved method based on CBAM-BCEL1-INGAN for augmenting side-scan sonar seafloor sediment image datasets. This method incorporates an attention mechanism into the residual blocks of the generator to enhance the learning of specific attributes and improve the quality of the generated images. Additionally, the BCEL1 loss function is introduced into the discriminator, allowing it to focus on both global image consistency and finer distinctions. The aim is to effectively utilize existing data to expand the dataset and enhance the performance and generalization ability of deep learning models. Experimental results demonstrate that the proposed method for augmenting side-scan sonar seafloor sediment image datasets can learn features of various sediment sonar images and generate a large number of augmented samples, providing an effective solution to the problem of the lack of diversity and quantity of side-scan sonar seafloor sediment image data samples.

## 2. Materials and Methods

### 2.1. InGAN Model

**Figure 1:**
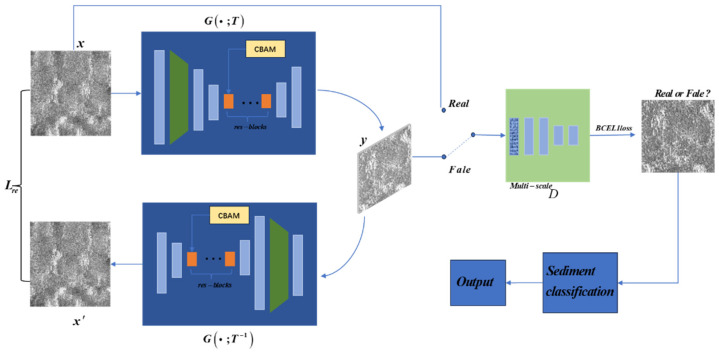
CBAM-BCEL1-INGAN network flowchart.

The purpose of InGAN is to learn the internal blocks of images rather than making structural and stylistic transformations to the images. However, using the INGAN network to train side-scan sonar seafloor sediment images tends to generate unrealistic images. Given the distinct grayscale and texture features of side-scan sonar images, this paper proposes a method for augmenting side-scan sonar seafloor sediment image datasets based on CBAM-BCEL1-INGAN. The specific network process is illustrated in Figure 1. Firstly, an attention mechanism is incorporated into the residual blocks of the INGAN network generator to enhance the learning of specific attributes and improve the quality of the generated images. Secondly, the discriminator introduces the BCEL1 loss function (a combination of binary cross-entropy loss and L1 loss). This allows the discriminator to focus on both global image consistency (L1 loss) and finer distinctions (binary cross-entropy loss), thereby achieving better generation results. Finally, the augmented samples are added to the test set for sediment classification in order to confirm their authenticity.

The InGAN model consists of a generator G and a multiscale discriminator D. The generator redirects input x to output y, whose size or shape is determined by a geometric transformation T. The multiscale discriminator D learns to distinguish the block statistics of the fake output y from the real block statistics of the input images. Additionally, by leveraging the self-homomorphism of G and by using G and its inverse transformation T-1, we reconstruct input x from y. This introduces a concept similar to “cycle consistency”, where y generated from x is fed back into the generator to reconstruct the original scale image x’, ideally making x and x’ identical.

However, this process differs fundamentally from approaches such as CycleGAN [17,18], which involve dual generators and discriminators: one for A → B and another for B → A transformations. In contrast, InGAN operates with a single pathway, focusing on learning internal blocks of images without structural or stylistic transformations.

**Figure 2:**
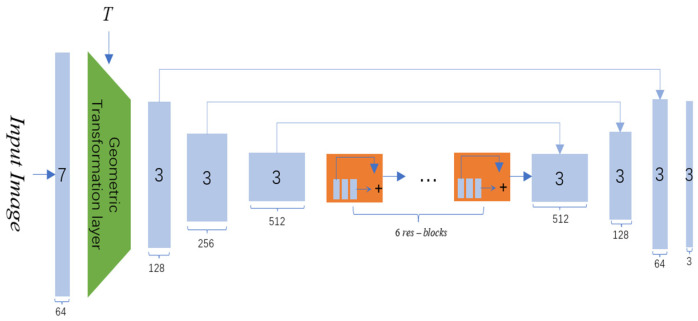
Generator structure diagram.

The generator in InGAN comprises three parts: convolutional layers for up or downsampling and image feature extraction, geometric transformation layers for image scale transformation, and residual layers for deepening image feature extraction. The structure of the generator is illustrated in Figure 2.

**Figure 3:**
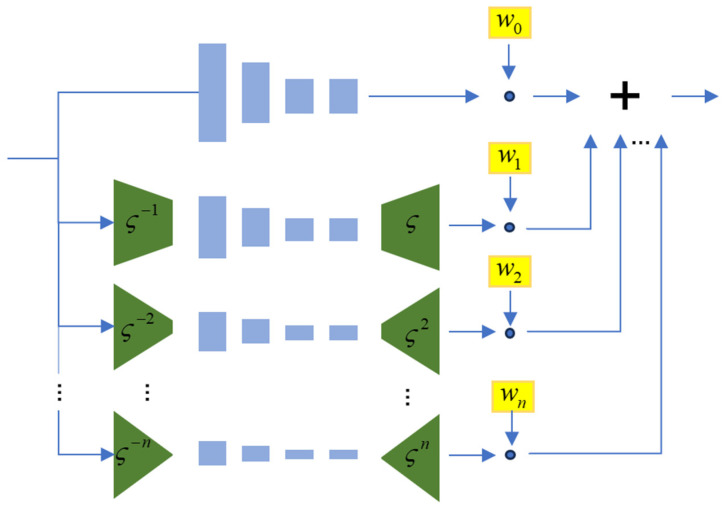
Multiscale discriminator.

In the optimization phase, as shown in Figure 3, discriminator D is a multiscale discriminator. It evaluates the authenticity of generated images at different scales by comparing them with real images, weighting the scores obtained from the different scales to optimize the adversarial loss. This approach enhances the control and stability of the GAN in generating high-quality images.

The discriminator employs a fully convolutional structure, as depicted in the figure. At a single scale, it typically consists of four convolutional layers: a convolutional extraction layer, followed by a downsampling layer, a conventional convolutional layer, and, finally, a sigmoid activation layer producing scores in the range [0, 1]. This setup analyzes the structure of the image at that scale to determine authenticity.

For a multiscale evaluation, the discriminator computes weighted scores for each scale and aggregates them to produce the final output. Scale weights are designed to enable multiscale discrimination, enhancing the model’s ability to analyze images across different resolutions effectively.

(1) n=logςInputSizeReceptiveField

(2) $$\sum_{i=0}^{n}w_{i}=1$$

where *n* represents the number of scales or layers in the multiscale discriminator; ς indicates the downsampling factor; InputSize denotes the spatial resolution (number of pixels) of the input image at scale *n*; ReceptiveField represents the effective field of the discriminator, indicating the range over which each position in the network receives input; and $w_{i}$ signifies the weights allocated to each scale, used to weight the outputs of the discriminator at different scales to obtain the final discriminative result.

The generator uses the geometric transformation $T^{-1}$ and the forged *y* to obtain the reconstructed version of *x*′. The optimization method similar to LSGAN [19] is as follows:

(3) $$L_{GAN}\left(G,D\right)=E_{y∼P_{data}\left(x\right)}\left[{\left(D\left(x\right)-1\right)}^{2}\right]+E_{x∼P_{data}\left(x\right)}\left[D{\left(G\left(x\right)\right)}^{2}\right]$$

where *x* represents a real sample, D(x) indicates the score given to the real sample by the discriminator. A score closer to 1 means that the discriminator considers the sample more realistic. G(x) represents a sample generated by the generator based on *x*, which follows the same distribution. D(G(x)) is the score given by the discriminator to the generated sample. If D considers the generated sample more fake, the score D(G(x)) will be closer to 0.

The reconstruction process is as follows: first, *y* = *G* (*x*; *T*), and then *x*′ = *G* (*y*; $T^{-1}$). The reconstruction loss is as follows:

(4) $$L_{re}={\left\|G\left(G\left(x;T\right);T^{-1}\right)-x\right\|}_{1}$$

Through the loss of the confrontation process and the reconstruction process, the final loss function of InGAN is obtained as follows:

(5) $$L_{INGAN}=L_{GAN}+\lambda \cdot L_{re}$$

### 2.2. Residual Block Based on CBAM

**Figure 4:**
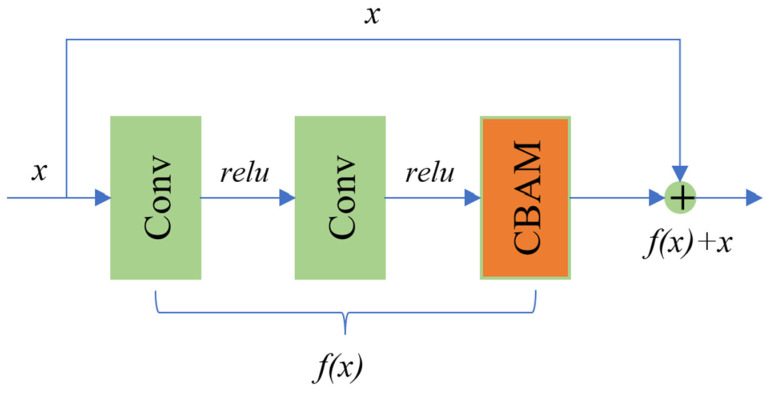
Residual block structure based on CBAM.

In the research using GANs to augment side-scan sonar image data, it is crucial to fully learn the background and texture features of the images. In the generator of the INGAN network, six residual layers are added to deepen the network. In this study, to enhance the generator’s learning of specific attributes and improve the quality of the generated images, a Convolutional Block Attention Module (CBAM) is incorporated into the residual blocks, as illustrated in Figure 4.

The CBAM [20] consists of two submodules: channel attention and spatial attention. Channel attention is used to adjust the importance between different channels of the feature map, while spatial attention is used to adjust the importance between the spatial positions of the feature map. The combination of these two submodules can make the model more focused on important features and help to improve its generalization ability and performance.

In CBAM-based residual blocks, features are first extracted through convolution operations. Then, the CBAM is used to adjust the channel attention and spatial attention to these features. Finally, the adjusted features are residually connected to the input in order to retain the information of the original features and enhance the representation of important features.

In the residual block, two convolution layers are used to extract the features of the input tensor, the attention mechanism is used to adjust the features to make the features more prominent, and then the attention-adjusted features are added to the original input tensor to form the final output. The aim is to prevent the problems of increasing training difficulty and information loss when training the deep network.

### 2.3. Discriminator Based on BCEL1 Loss Function

In our study, we choose binary cross-entropy loss and L1 loss as loss functions in the generative adversarial network (GAN). These two loss functions each provide effective optimization targets for different aspects of the GAN. Binary cross-entropy loss is mainly used in discriminator training. It measures the difference between the real sample and the generated sample based on the log-likelihood principle, and it enables the discriminator to distinguish the two more accurately. Specifically, the binary cross-entropy loss function (*BCE*) is as follows:

(6) $$BCE\left(D\left(x\right),y\right)=-\frac{1}{N}\sum_{i=1}^{N}\left[y_{i}\log\left(\sigma \left(D\left(x_{i}\right)\right)\right)+\left(1-y_{i}\right)\log\left(1-\sigma \left(D\left(x_{i}\right)\right)\right)\right]$$

where *y* is a binary label (0 or 1) representing the true class of the sample and $D\left(x\right)$ denotes the output of the discriminator, which represents the probability that the sample is real.

*L*1 loss, in contrast, focuses on pixel-level differences in the generated image, helping to maintain structural and semantic consistency between the generated image and the target image. The *L*1 loss function is as follows:

(7) $$L1(G\left(z\right),t)=\frac{1}{N}\sum_{i=1}^{N}\left|G\left(z_{i}\right)-t_{i}\right|$$

where $G\left(z\right)$ is the generated image, *t* is the target image, and *N* is the number of pixels.

The reason for choosing these two loss functions is that they emphasize different aspects of the adversarial generation network, allowing the model to optimize the quality and consistency of the generated images more comprehensively. However, due to the different measurement scales of these two loss functions, directly adding them together may result in one loss function contributing excessively to the total loss. To avoid this issue, the total loss function is defined as the comparison and sum of the two loss functions, with normalization applied to both loss functions.

(8) $$BCE_{n}=\frac{BCE}{BCE_{init}}$$

(9) $$L1_{n}=\frac{L1}{L1_{init}}$$

(10) $$TotalLoss=\frac{1}{2}\left(BCE_{n}+L1_{n}\right)$$

where $BCE_{init}$ and $L1_{init}$ are the initial values of the binary cross-entropy loss function and the *L*1 loss function, respectively. By using the above method, both loss functions can maintain a similar scale during the training process, thus avoiding bias towards one particular loss function.

## 3. Experimental and Results

Based on the CBAM-BCEL1-INGAN framework, augmenting the seabed sediment images obtained from side-scan sonar data is a crucial component of this study. To evaluate the feasibility and effectiveness of our approach, we assess the performance of the proposed GAN method. Firstly, qualitative and quantitative analyses are conducted on the quality of the generated images. Subsequently, through ablation experiments, we qualitatively and quantitatively analyze the effectiveness of the strategies employed within the GAN. Finally, a trained classification model is utilized to predict the classes of the generated images, validating their effectiveness.

**Figure 5:**
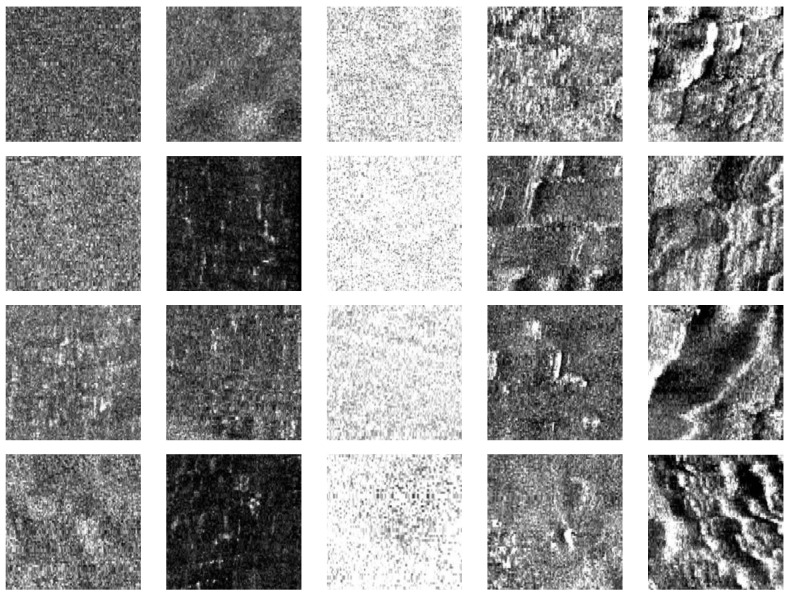
Parts of the samples in the dataset.

The dataset used in our experiments consists of side-scan sonar data collected in 2019 from the Jiaozhou Bay area, Qingdao, using a Klein4000 side-scan sonar system manufactured by Klein Corporation of United States. We selected representative images depicting mud-sand, sandy mud, fine sand, coarse sand, gravel, and bedrock sediment types for experimentation. Examples of some sample images are shown below, see Figure 5.

### 3.1. Evaluation Index

For the generated image, we mainly consider two factors, namely, the sharpness and the diversity, while also considering the similarity between the generated image and the real image; therefore, in this study, the Frechet Inception Distance (*FID*), Kernel Maximum Mean Discrepancy (MMD), Inception Score (IS), Peak Signal-to-Noise Ratio (PSNR), and Structural Similarity (SSIM) are selected as evaluation indicators to analyze the quality of the generated images.

*FID* is an indicator used to assess the quality and variety of images generated by a generative model. It works by comparing the distribution of the generated image and the real image in a specific space, and it represents the distance between the feature vector of the generated image and the feature vector of the real image. The calculation formula is

(11) $$FID={\left\|\mu_{g}-\mu_{r}\right\|}_{2}^{2}+Tr\left(\sum_{g}+\sum_{r}-2{\left(\sum_{g}\sum_{r}\right)}^{1/2}\right)$$

where ${\left\|\mu_{g}-\mu_{r}\right\|}_{2}^{2}$ is the *L*2 norm of the square of the mean vector difference; Tr represents the trace of the matrix (i.e., the sum of the diagonal elements of the matrix); and ${\left(\sum_{g}\sum_{r}\right)}^{1/2}$ is the square root of the product of the covariance matrix $\sum_{g}$ and $\sum_{r}$, representing the matrix obtained by taking the square root of the eigenvalues of the product of two matrices.

According to the formula, it can be observed that the greater the difference in the mean vector between the generated and sample images, the larger the FID value. The main objective of this study is to ensure that single-image augmentation methods generate diverse images. This diversity allows for the generated images to possess characteristics similar to those of the sample images while maximizing the difference in the mean vector from the sample images. Therefore, a larger FID value indicates greater diversity in the generated images.

The *MMD* is the maximum average difference between two distributed samples that are small enough to consider the two distributions the same; otherwise, they are considered different. In image generation, the lower the *MMD* value, the more realistic the generated image. The calculation formula is

(12) $$MMD^{2}\left(P_{r},P_{g}\right)=E_{x_{r},{x_{r}}^{\prime }∼P_{r},x_{g},{x_{g}}^{\prime }∼P_{g}}\left[k\left(x_{r},{x_{r}}^{\prime }\right)-2k\left(x_{r},x_{g}\right)+k\left(x_{g},{x_{g}}^{\prime }\right)\right]$$

where $x_{r}$ is the source domain data, and $x_{g}$ is the target domain data, which measures the difference between the real distribution $P_{r}$ and the generated distribution $P_{g}$ given a fixed kernel function *k*.

The Inception Score is a metric used to measure the sharpness and variety of the generated images.

(13) $$IS\left(G\right)=\exp\left(E_{x}D_{KL}\left(p\left(y|x\right)∥p\left(y\right)\right)\right)$$

where $p\left(y|x\right)$ represents the probability distribution of the generated images belonging to each category, and $p\left(y\right)$ represents the probability distribution of the label vectors obtained from the generated samples.

The *PSNR* is a vital measure in image quality assessment and is widely used in image processing. In image denoising, the noise power is determined by computing the Mean Squared Error (*MSE*) between the denoised image and the original image. For an original image (*I*) and its denoised version (*R*), the *MSE* is computed as follows:

(14) $$MSE=\frac{1}{MN}\;\sum_{i=1}^{M-1}\sum_{j=1}^{N-1}{\left[I(i,j)-R(i,j)\right]}^{2}$$

The *PSNR* is defined as

(15) $$PSNR=10\log_{10}(\frac{Max_{I}^{2}}{MSE})$$

where $Max_{I}^{2}$ represents the maximum pixel value of the original image. In image denoising research, a higher *PSNR* indicates that the denoised image *R* contains less noise, thus implying better denoising effectiveness.

Structural Similarity (*SSIM*) is a metric used to gauge image similarity, and it is also applicable for assessing compressed image quality. This study utilizes *SSIM* to evaluate denoised image quality by computing the Structural Similarity between the denoised and original images. *SSIM* assesses image similarity based on three factors: luminance, contrast, and structure.

Given two input images, *x* and *y*, the definition of *SSIM* is as follows:

(16) $$SSIM(x,y)={\left[l(x,y)\right]}^{\alpha }{\left[c(x,y)\right]}^{\beta }{\left[s(x,y)\right]}^{\gamma }(\alpha ,\beta ,\gamma >0)$$

With

(17) $$l(x,y)=\frac{2\mu_{x}\mu_{y}+c_{1}}{{\mu_{x}}^{2}+{\mu_{y}}^{2}+c_{1}}$$

(18) $$c(x,y)=\frac{2\sigma_{xy}+c_{2}}{{\sigma_{x}}^{2}+{\sigma_{y}}^{2}+c_{2}}$$

(19) $$s(x,y)=\frac{\sigma_{xy}+c_{3}}{\sigma_{x}+\sigma_{y}+c_{3}}$$

where $l\left(x,y\right)$ is the brightness comparison, $c\left(x,y\right)$ is the contrast comparison, and $s\left(x,y\right)$ represents the structure comparison. $\mu_{x}$ and $\mu_{y}$ denote the mean values of *x* and y, respectively, while $\sigma_{x}$ and $\sigma_{y}$ represent the standard deviations of *x* and *y*, respectively. $\sigma_{xy}$ denotes the covariance between *x* and *y*, and $c_{1},\;c_{2},$ and $c_{3}$ are constants used to avoid division by zero errors.

In practical calculations, it is common to set α=β=γ=1 and $c_{3}=c_{2}/2$. This simplifies the definition of *SSIM* to

(20) $$SSIM(x,y)=\frac{\left(2\mu_{x}\mu_{y}+c_{1})(\sigma_{xy}+c_{2}\right)}{\left({\mu_{x}}^{2}+{\mu_{y}}^{2}+c_{1})({\sigma_{x}}^{2}+{\sigma_{y}}^{2}+c_{2}\right)}$$

*SSIM* ranges from 0 to 1, with a higher value indicating less disparity between the output image and the undistorted image, indicating superior image quality. When two images are identical, *SSIM* equals 1.

### 3.2. Experimental Design

To verify the effectiveness of the CBAM-BCEL1-INGAN side-scan sonar image dataset amplification method proposed in this paper, the side-scan sonar images of six types of sediment—muddy sand, sandy mud, fine sand, coarse sand, gravel, and bedrock—were amplified, and images with representative characteristics were selected for training. New natural images of different sizes were generated. The model training was implemented using Python language based on the Pytorch framework, and the hardware environment was as follows: the operating system was Windows 11; the CPU was 12th Gen Intel (R) Core (TM) *i9-12900H 2.50GHz*; the GPU was one *NVIDIA GeForce RTX3050*; and the memory was *4GB*.

#### 3.2.1. Validity Verification of Amplified Images

To verify the effectiveness of substrate image amplification using this method, representative images of different substrates were selected for training. Some amplification samples are shown in Table 1 below.

The FID, MMD, IS, PSNR, and SSIM indices were calculated to evaluate the image quality of nine amplified samples of the above six types of substrates.

When the batch image is amplified, the smaller the FID value, the higher the quality and diversity of the generated image, but, for single-image amplification, the smaller the FID value, the closer the generated image to the original image; thus, in the process of single-image amplification, a larger FID value indicates that the generated image has higher quality and diversity. An analysis of Table 2 shows that coarse sand, gravel, and bedrock have high FID values, indicating that these three substrates have better effects in the single-image substrate amplification experiment. The MMD values of all categories are around 1.02, which indicates that there is a certain similarity between the generated image and the real image in terms of feature statistics. Regarding value size, there is no significant difference between several substrates. Regarding the IS index, the larger the value, the better the clarity and diversity of the generated image, and an analysis of the value shows that the three substrates of coarse sand, gravel, and bedrock have better effects. By analyzing the PSNR and SSIM metrics, it is found that the PSNR values for all categories are relatively low, and the SSIM values are close to 0. This indicates that the generated images have a low similarity to the original images, which is sufficient to demonstrate the diversity of the generated images.

For the above analysis, the entropy of the gray co-occurrence matrix is used for a quantitative analysis. When all values in the co-occurrence matrix are equal, or the pixel values show the greatest randomness, the entropy is the highest. Therefore, the entropy value indicates the complexity of the gray distribution of the image. The higher the entropy value, the more complex the image. It can be seen from the entropy value in Table 2 that the entropy of the three substrates, coarse sand, gravel, and bedrock, is larger.

#### 3.2.2. Ablation Experiment and Evaluation

The role of each module in the performance of this model was verified. Ablation experiments were conducted on the CBAM and BCEL1 loss function by using the control variable method, and the evaluation indices were the FID, MMD, IS, PSNR, and SSIM. Four groups of control experiments were designed with the bedrock 1 image as the experimental object, and the experimental results are shown in Table 3. By comparing Groups 1 and 2, it can be seen that the quality of the images generated by the model after incorporating the attention mechanism is higher, which proves the effectiveness of the residual block based on the CBAM proposed in this paper for the model. By comparing Groups 3 and 1, we can see the superiority of the BCEL1 loss function proposed in this paper. By comparing Group 4 with Groups 2 and 3, it can be seen that the model with the combination of the CBAM and BCEL1 loss function has better performance than that with only a single strategy, indicating that the combination plays a crucial role in improving the overall performance of the model, thus reflecting the effectiveness of the method proposed in this paper.

**Figure 6:**
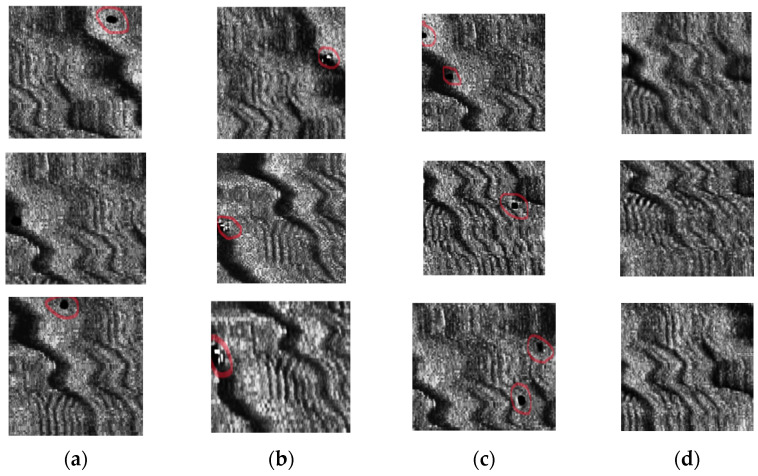
Amplification of 4 groups of models, Figures (**a**–**d**) are partial amplifications generated by models 1–4 (The red circles are features of unnecessary detail).

The partial amplification of bedrock 1 images by the four groups of models trained with different strategies is shown in Figure 6. By comparing Models 2 and 1, it can be seen that the model with the CBAM can improve the quality and diversity of the generated images in terms of the evaluation indicators, but some images show unnecessary details. By comparing Models 3 and 1, it can be seen that the model using the BCEL1 loss function can achieve better image generation, but it also produces unnecessary defects while improving the index. By comparing Models 4 and 1, it can be seen that the model combining the CBAM and BCEL1 loss function performs well in terms of the evaluation indicators and the image texture, edge, and other details, which proves the effectiveness of the proposed method.

#### 3.2.3. Classification Model Verification

Considering that the purposes of this study are to expand side-scan sonar substrate images so as to improve the performance of deep learning-based substrate classification models and to expand the training set because it contains a small number of samples, a deep learning-based object detection model is selected for comparative experiments. At present, there are many detection models, and the AlexNet network, as a lightweight, fast, and mature detection model, is suitable for this experiment. Therefore, the AlexNet network is selected to train real images, obtain a classification model, and classify the generated images.

The training and testing datasets consist of side-scan sonar data collected in 2019 from the Jiaozhou Bay area in Qingdao, utilizing a Klein4000 side-scan sonar system. There are a total of 27,202 sonar images across five different sediment types. These include 5123 images of sandy mud sediment, 5851 images of muddy sand sediment, 7126 images of fine sand sediment, 4406 images of gravel sediment, and 4696 images of bedrock sediment. For detailed information about the image library, please refer to Table 4.

Currently, there are many detection models. This article uses five common models—AlexNet, GoogleNet, VggNet, ResNet, and DenseNet—to train the dataset, and the hyperparameter selection is shown in Table 5.

The training accuracy curve and training time are shown in Table 6.

**Figure 7:**
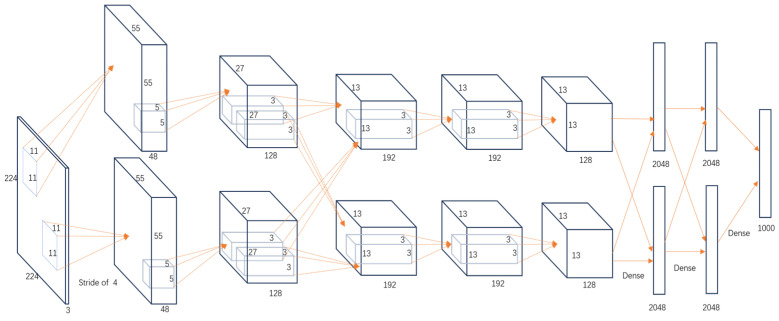
Structure of the AlexNet network model (The direction the arrow points in indicates the path of data from one layer to the next).

It is evident from the above training results that the AlexNet network has the shortest training time, and the convergence value of the validation accuracy curve is the highest, at approximately 92%, making it more suitable for this experiment. The AlexNet network consists of approximately 630 million connections, and it includes five convolutional layers and three pooling layers. It utilizes fully connected layers and a softmax layer for image classification, as shown in Figure 7. Each convolutional layer consists of convolutional kernels, bias terms, rectified linear unit activation functions, and local response normalization modules. The first, second, and fifth convolutional layers are followed by a max pooling layer, and the last three layers are fully connected layers. The final output layer is a softmax layer, which converts the network output into the probability values used for predicting the image’s class [21].

By using the AlexNet network model trained on real side-scan sonar substrate images, we randomly selected 100 images from the augmented images of bedrock image 1 and the real image test set in Section 3.2.2 for validation. The validation was conducted 10 times, and the average value was taken. The specific details are shown in Table 7.

An analysis of Table 7 shows that both the model with only the CBAM (Group 2) and the model using only the BCEL1 loss function (Group 3) improve the accuracy of the generated images compared with Group 1. However, the model that integrates the CBAM and the BCEL1 loss function achieves the greatest accuracy improvement, with an increase of 6.8% compared with Group 1. Additionally, it still maintains a high recognition rate when tested against the real image dataset. The above experiments demonstrate that the images generated using the method proposed in this paper are closer in realism to the actual side-scan sonar substrate images.

Due to the limited sampling points of coarse sand in the training set, six types of substrate sonar images were selected from the original dataset, and several were chosen from the generated images. Three groups of datasets were designed to train the AlexNet network: one dataset containing only the original images, one dataset containing both the original images and the images generated using the proposed augmentation method, and one dataset containing both the original images and images augmented using simple methods (such as flipping and rotating). The specific details can be found in Table 8.

The dataset is divided into training and validation sets in a ratio of 2:1, and 100 real images of bedrock substrate are selected from the original images to test the model’s performance. The results are shown in Table 9.

Table 9, comparing the detection results of groups 1 and 2, shows that the classification accuracy of bedrock-type matrices is improved by 5.2% when images enhanced using the method presented in this paper are added to the training set. In contrast, a comparison of groups 1 and 3 showed that the accuracy was only 2.5% better when images enhanced with a simple method were added to the training set. The reason for this is that images enhanced with a simple method do not increase the diversity of the base image simply because the increase in number improves the accuracy. This indicates that the improvement of the model performance is mainly due to the use of the enhanced data generated using the method proposed in this paper, which further indicates that the enhanced image meets the requirements of the realism and diversity of the side-scan sonar image.

## 4. Discussion

In this paper, the CBAM-BCEL1-INGAN method based on side-scan sonar shows good results in image generation tasks of different bottom types. Through the experimental analysis and result evaluation in this paper, the following conclusions are drawn:

(1) The images generated using the method proposed in this paper perform well in terms of quality and diversity. In particular, for sediment types such as coarse sand, gravel, and bedrock, the images generated show high quality and diversity in evaluation indicators such as the FID value and IS index. This shows that the proposed method can effectively enlarge the images of these complex substrates, enrich the dataset, and improve the generalization ability of the model. (2) Through an entropy analysis of the gray co-occurrence matrix, the complexity and authenticity of the generated image are further verified. The larger the entropy, the more complex the gray distribution of the image. The entropy of the coarse sand, gravel, and bedrock images produced in this study is high, which proves that these images are close to the real images in terms of detail and texture and have high complexity and naturalness. (3) Ablation experiment results show that both the CBAM and BCEL1 loss functions contribute significantly to the quality improvement of the generated images. Specifically, the introduction of the CBAM improves the attention mechanism of the model and enriches the details of the generated images. Although the BCEL1 loss function restricts image generation, it improves the overall quality and consistency of the generated image. The model with the fusion of these two modules shows the best performance on all evaluation indices, which verifies the effectiveness and superiority of the proposed method in the image generation task. (4) In the verification experiment of the classification model, the AlexNet classification model is used to test the accuracy of the images generated using the proposed method, and it is concluded that the image generated using the model combining the CBAM and BCEL1 loss function has the highest recognition rate. This further proves that the proposed method can generate high-quality amplified images.

**Figure 8:**
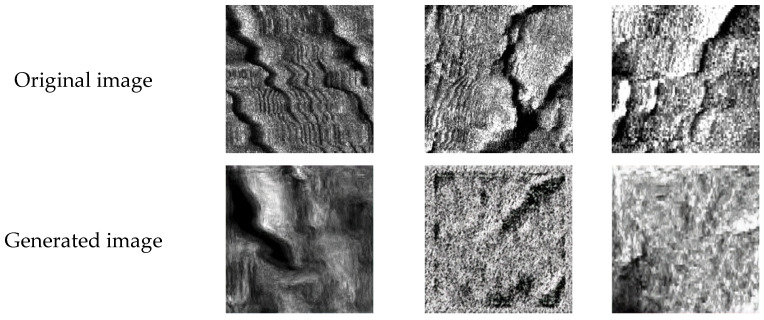
Some images were generated using the sinGAN network during training.

In addition to the INGAN network used in this paper, the sinGAN [22] network, has achieved good results in the field of image augmentation. However, its performance in augmenting side-scan sonar substrate images is not ideal. As shown in Figure 8, some images generated using the sinGAN network during training have lost the characteristics of the original images, and some of them are no longer similar to the side-scan sonar images.

The method proposed in this paper has significant value in practical applications. The acquisition of side-scan sonar images is costly and challenging, while the method presented here can effectively augment the dataset, reducing the reliance on a large number of real images and lowering costs. Specifically, the generated high-quality images can be used for the following practical seabed exploration tasks: (1) Training more robust models: The generated high-quality images can be used to train more robust seabed substrate classification models, thereby improving the accuracy and reliability of the models in practical applications. This is of great significance for automated seabed exploration and resource assessment. (2) Applications in data-scarce environments: In situations where it is difficult to obtain large-scale real data, the generated images can serve as supplementary data, effectively addressing the issue of data scarcity. For example, in deep-sea exploration, obtaining a large number of high-quality sonar images is extremely difficult due to harsh environments and high costs. The method proposed in this paper can significantly reduce the reliance on actual collected data. (3) Reducing data annotation costs: By using the generated images, the demand for manual annotation can be reduced, thereby lowering data annotation costs. This provides a cost-effective solution, especially in machine learning tasks that require a large amount of annotated data.

In summary, the CBAM-BCEL1-INGAN image amplification method proposed in this paper has excellent performance in the amplification task of side-scan sonar submarine bottom images, and its effectiveness and superiority are verified by several experiments. Future studies can further optimize the model structure and training strategies, explore more amplification methods suitable for different application scenarios, and further improve the quality and diversity of the generated images.

## 5. Conclusions

Aiming to address the problems of difficulty in acquiring seafloor sediment image datasets, high labeling costs, and the insufficient diversity and quantity of data samples, this paper proposes a method for the sample amplification of seafloor sediment images obtained using side-scan sonar based on CBAM-BCEL1-INGAN. A residual block based on the CBAM is designed to retain information about the original features and enhance the representation of important features. The BCEL1 loss function is designed based on the original L1 loss function so that the discriminator can pay attention to both the global image consistency and more subtle differences at the same time in order to obtain a better generation effect. Through experiments on existing sediment image datasets, it is confirmed that the proposed method performs well in the task of sediment image generation, and it solves the problem of the lack of side-scan sonar sediment image data to a certain extent. The images generated using the method proposed in this paper can be effectively used for practical underwater exploration tasks, reducing the reliance on a large number of real images, lowering costs, and improving the accuracy and reliability of seabed substrate classification and other related tasks. This not only enriches the dataset but also significantly reduces the costs of data acquisition and labeling. Future research can further optimize the model structure and training strategies to explore more augmentation methods suitable for different application scenarios, further enhancing the quality and diversity of the generated images.

## Figures and Tables

**Table 1:** Partial amplification examples.

Substrate	Training Sample	Generated Sample			
Muddy sand	** 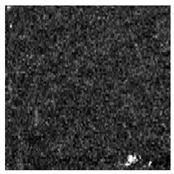 **	** 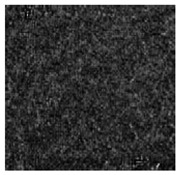 **	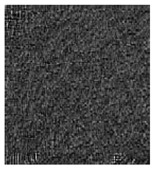	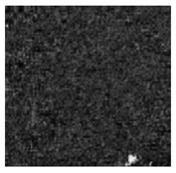	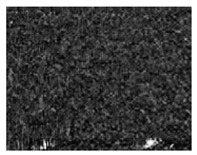
Sandy mud	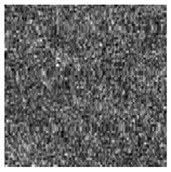	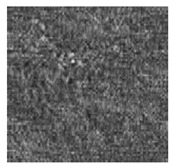	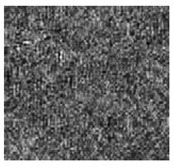	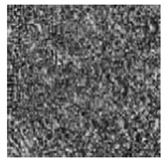	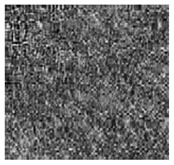
Fine sand	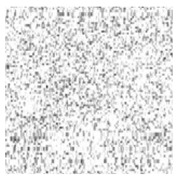	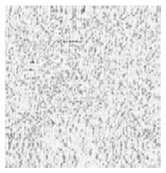	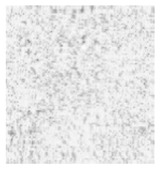	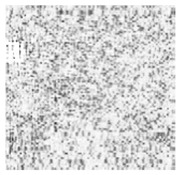	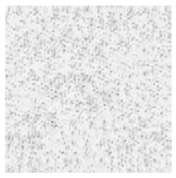
Coarse sand	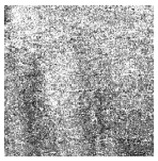	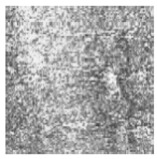	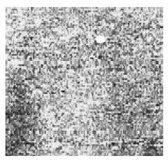	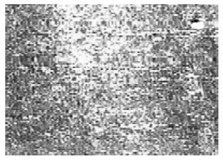	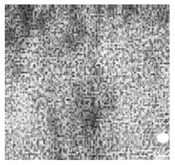
Gravel 1	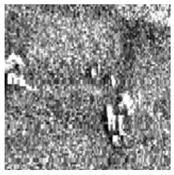	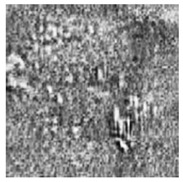	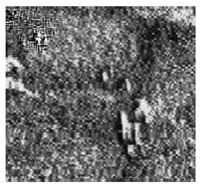	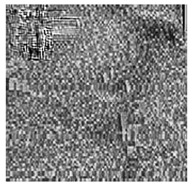	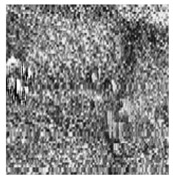
Gravel 2	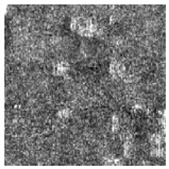	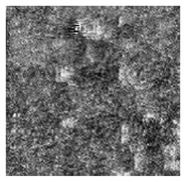	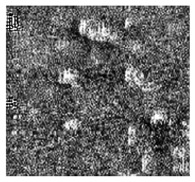	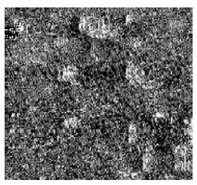	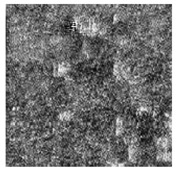
Bedrock 1	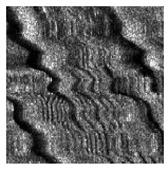	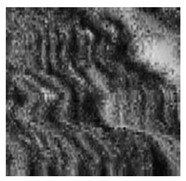	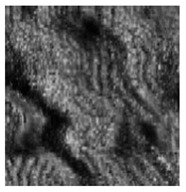	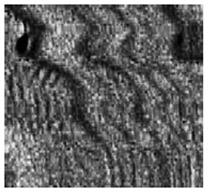	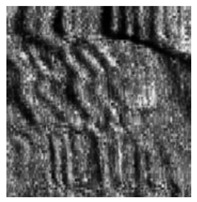
Bedrock 2	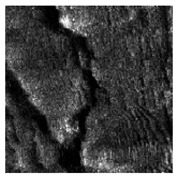	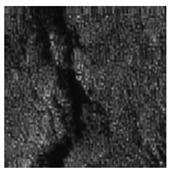	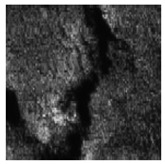	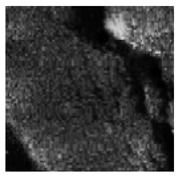	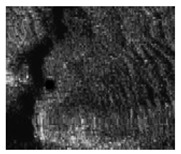
Bedrock 3	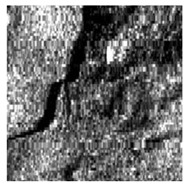	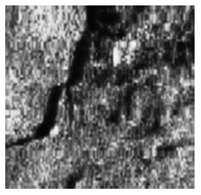	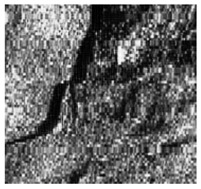	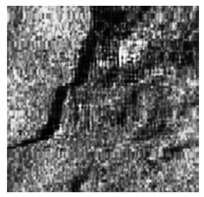	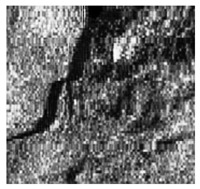

**Table 2:** Image quality evaluation.

Group	Entropy of Gray Co-Occurrence Matrix	FID	MMD	IS	PSNR	SSIM
Muddy sand	11.9461	448.58	1.023	1.2474 ± 0.1040	14.32	0.19
Sandy mud	13.2604	403.44	1.022	1.2379 ± 0.0602	9.52	0.06
Fine sand	11.6167	284.60	1.017	1.1847 ± 0.1110	9.44	0.11
Coarse sand	14.2414	902.46	1.017	1.6031 ± 0.2075	6.67	0.02
Gravel 1	13.8789	1001.79	1.020	1.4307 ± 0.1345	8.12	0.10
Gravel 2	13.5145	686.24	1.020	1.4044 ± 0.1315	10.19	0.09
Bedrock 1	13.4833	1024.62	1.033	1.4070 ± 0.1720	6.49	0.03
Bedrock 2	12.3735	987.43	1.020	1.4499 ± 0.1658	10.13	0.09
Bedrock 3	13.8416	576.86	1.015	1.3268 ± 0.0914	9.00	0.18

**Table 3:** Network performance of different methods.

Model	CBAM Model	BCEL1 Loss	FID	MMD	IS	PSNR	SSIM
1	—	—	877.12	1.035	1.2711 ± 0.1176	7.10	0.069
2	—	√	895.29	1.033	1.3861 ± 0.1671	7.03	0.065
3	√	—	944.60	1.033	1.3667 ± 0.0935	7.25	0.066
4	√	√	1024.62	1.033	1.4070 ± 0.1720	6.44	0.058

**Table 4:** A side-scan sonar image library was used in the experiment.

	Sandy Mud	Muddy Sand	Sand	Gravel	Bedrock
Example diagram	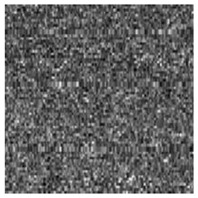	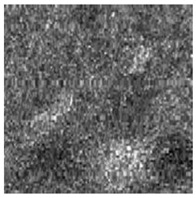	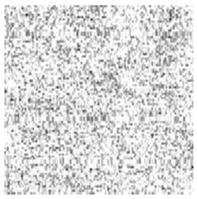	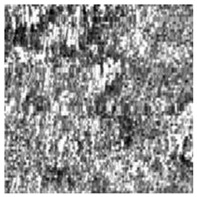	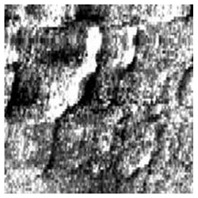
	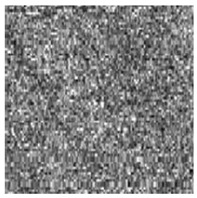	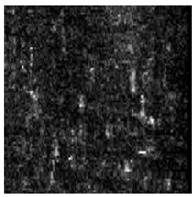	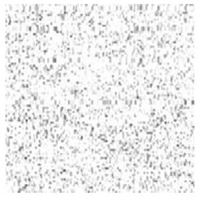	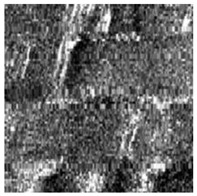	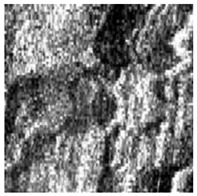
	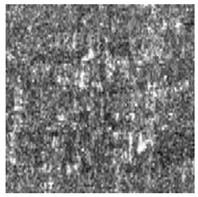	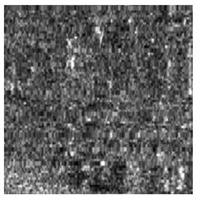	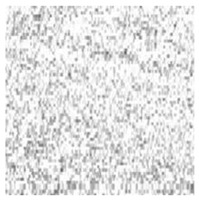	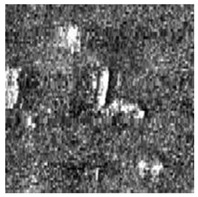	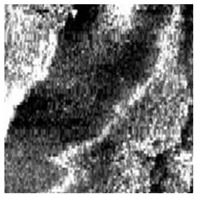
	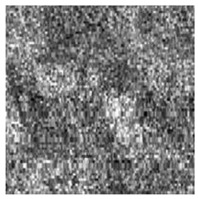	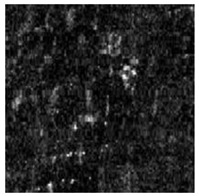	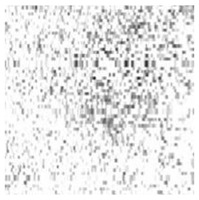	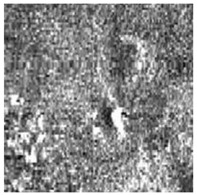	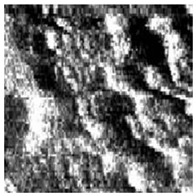
Quantity	5123	5851	7126	4406	4696
Total	27,202				
Size	256 × 256				

**Table 5:** Basic parameter settings of the network.

Training Parameters	Parameter Settings
Training Epochs	100
batch_size	32
Learning Rate	0.0001

**Table 6:** Training results of five models.

Model	Training Duration (min)	Validation Accuracy Curve
AlexNet	121.8	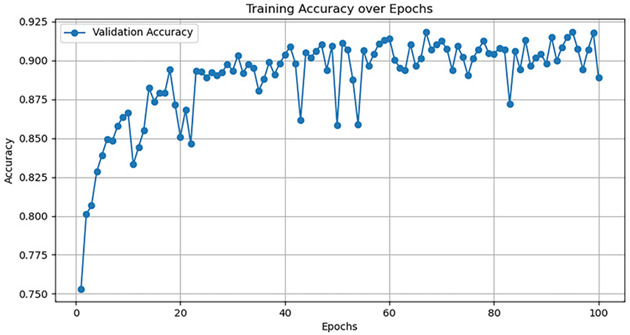
GoogleNet	129.1	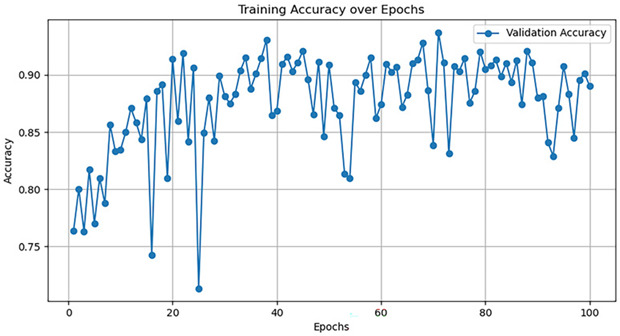
VggNet	161.9	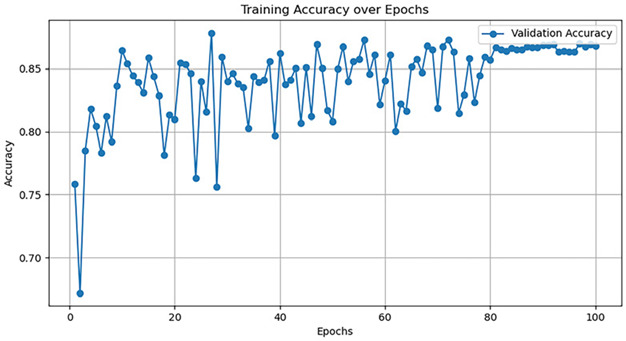
ResNet	124.3	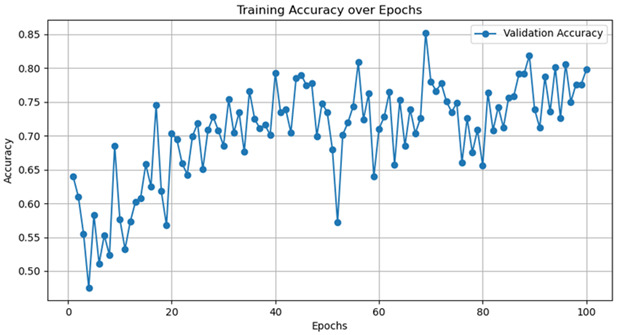
DenseNet	135.6	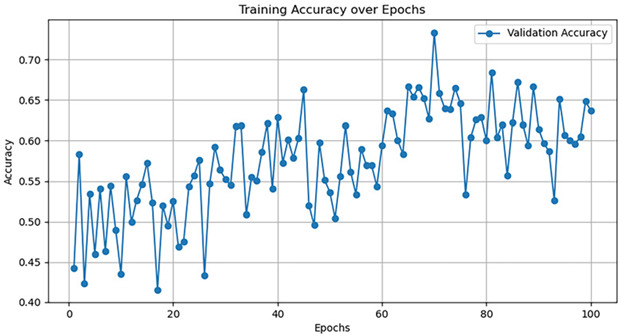

**Table 7:** Detection results of the generated images using the classification model.

Group	CBAM Model	BCEL1 Loss	Generated Image Bedrock Recognition Rate
1	—	—	90.50%
2	—	√	93.60%
3	√	—	92.20%
4	√	√	97.30%
5	Original dataset		92.60%

**Table 8:** Composition of the three groups of datasets.

Group	Original Image	Images Augmented Using the Proposed Method	Images Augmented Using the Simple Method
1	260	—	—
2	200	60	—
3	200	—	60

**Table 9:** Detection results of real bedrock substrate images with different training sets.

	Accuracy
AlexNet-1	82.50%
AlexNet-2	87.70%
AlexNet-3	85.00%

## Data Availability

Sample data are included in the article.
